# Evaluating the Role of Support Group Structures as Vehicles of Palliative Care: Giving Productivity in the Kanye Care Program in Botswana

**DOI:** 10.4103/0973-1075.78444

**Published:** 2011

**Authors:** Simon Kang’ethe

**Affiliations:** Department of Social Development, University of Fort Hare, P/B × 1314 Alice, 5700, RSA

**Keywords:** Community home-based care program, Palliative care giving, Palliative caregivers, People living with HIV/AIDS, Support groups

## Abstract

**Aims:**

The article aims to explore, evaluate, and discuss the role associated with the presence or absence of caregivers’ support groups in the Kanye care program in Botswana.

**Objective:**

The objective of the study was to solicit the views, attitudes, and thinking of palliative caregivers on the role, and implication of the presence and absence of support groups as vehicles influencing care-giving productivity.

**Materials and Methods:**

The study was explorative and descriptive in nature and used a qualitative design, involving 82 primary caregivers in focus group discussions and five community home-based care nurses in one- to-one interviews.

**Results:**

The support groups were found to play the following pivotal roles: (1) facilitate forums of strengthening caregivers; (2) facilitate sharing and exchange of information among caregivers; (3) facilitate counselling, debriefings, and education among caregivers; (4) foster the spirit and feelings of togetherness among caregivers; and (5) act as advocacy fronts and an opportunity of pooling and mobilizing the resources.

**Conclusion:**

The article recommends to the Government and the nongovernmental organizations to assist all care programs to institute and facilitate the formation of caregivers’ support groups.

## PROBLEM STATEMENT

Widespread concerns of the caregivers about lack of forums for encouraging one another and voice and share their concerns, griefs, debriefings, pains, and challenges associated with their care-giving occupations prompted the researcher to conduct the study and empirically assess the authenticity and the magnitude of these challenges in the care-giving arena and validate whether there were any support group structures to facilitate or provide a forum to address these challenges. Subjective information in the Kanye program was also perturbing, as some community members indicated that the program was a dumping process for the sick, that the Government was passing its responsibility of taking care of the sick to the community, and that institutional care was better. It was therefore justified and timely that an empirical and comprehensive study be carried out to either confirm the allegation or demystify the concern if found to be invalid.

## INTRODUCTION

The country of Botswana ranks highly among the countries hardest hit by the epidemic across the globe.[1] The situation has not improved much despite the heavy government investment in the HIV/AIDS campaign supported by the international community. This is partly evident from the 2008 Botswana AIDS Impact Survey III (BAIS III) results that were released in June 2009 that indicated a change in population based prevalence from 17.1% in 2004 to 17.6%. This shows an increase of 0.5% from year 2004 BAIS II.[2,3] Despite its small population of around 2 million people, the 2008 statistics indicated a population of 113,000 persons living with advanced HIV in the country.[4] This calls for various care mitigation factors to be put in place. It therefore remains an incontrovertible fact that social support remains one of the HIV/AIDS mitigation factors that need to be strongly mainstreamed in all the care-giving processes to give the caregivers and their clients adequate psychological, social, and emotional wellbeing (Nurses Association of Botswana, NAB).[5]

Taylor[6] defines social support as “information from others that one is loved and cared for, re-esteemed and valued….” Such information may come from a friend, a spouse, or other social networks such as churches, support groups, clubs, or health care workers who could have a strong remedial and bolstering impact upon the palliative caregivers and their care-giving productivity. Caregivers with a high level of social support can easily gain mastery and ownership over their care giving and can easily help clients cope well with their clinical diagnosis. A caregivers’ support group is considered important in empowering them to help their patients adopt better coping strategies and therefore overcome their diagnosis.[7] According to Kalanke,[8] these support groups are important to the caregivers of people living with HIV/AIDS (PLWHAs) because they ensure that information is availed and available about the nature of HIV/AIDS, causes of clients’ infection, the dates of infections, and what can be done to control the chances of re-infection and avoid full-blown AIDS. Support groups, therefore, strengthen and ensure that PLWHAs are taken good care of.

According to Kath Defilippi in Uys and Cameron,[9] a caregivers’ support group promotes teamwork and communication. A support group provides a forum for possible and easier professional intervention for caregiver support. According to Ferris *et al*.,[10] professional interventions for caregiver support are seen as fundamental to the maintenance of a healthy, caring organizational environment. Ferris *et al*.[10] acknowledge that primary caregivers who carry the lion’s share of burden in care giving need to be supported by being taught universal precautions, be encouraged to seek emotional support by talking to a friend and/or counsellor, be supported in seeking respite care to allow for a period of physical and emotional rest away from their care-giving responsibilities, and be informed of the need for, and be encouraged to pursue bereavement care following the death of their loved ones. This article explores, and evaluates the presence or absence of caregivers’ support groups and their influence on the care-giving productivity.

## STUDY LIMITATION

The research study from which this research article is derived was carried in 2005 and part of 2006. The article was written 3–4 years from then. The researcher, however, bears witness that not much has taken place as far as forming support groups in the area is concerned. The data and the article, therefore, remain scientifically relevant, valid, and informative.

## THEORETICAL FRAMEWORK

### Social network theory

The emphasis and importance of instituting caregivers’ support groups for care-giving effectiveness can be premised, grounded, or explained by the social network theory. According to Gottlieb,[11] social support is defined as “verbal and/or nonverbal information or advice, tangible aid, or action that is proffered by social inmates or inferred by their presence and has beneficial emotional or behavioural effects on the recipient.” The social network theory is important since it helps us to understand both the intrinsic and extrinsic benefits of why people interact with each other.[12] This is why Whittaker & Tracy[13] explain that “paradoxically, many of the families that are most in need of the social support that relatives, family and friends can provide are also the same families that are most isolated from relatives, family and friends… Other families are surrounded by social networks that are themselves beset by multiple problems.”

The social network theory advocates for helping clients to make an appropriate and effective use of social support by engaging them in the identification and assessment of potential social supports. This is the social network map.[12] In the case of social networks, which form this study’s interest, the objects or components of the network refer to people or caregivers who mutually and symbiotically come together to help one another, especially the one who critically needs to be helped to address a care-giving challenge by others.

An effective caregiver network will be an interaction between caregivers as they make debriefings to address the care-giving challenges that caregivers present. By understanding the mappings connecting one individual to others, or the social interactions, it is possible to evaluate, consider, and take stock of the social capital of a caregiver group and its individual members. Social capital refers to the network ability to draw on the resources contained by members of the network. To the caregivers, social capital is informed by the love, patience, and hope that different caregivers offer to one another.[14] Cooperation among the members is important and implies a significant exchange of sincere information between the members and some form of predisposition to help each other. This is to strengthen the cause and existence of the group dynamics.[15] For instance, those with care-giving skills such as palliative care, counselling, and nursing can benefit those without them, while those with leadership skills could help bolster leadership and advocacy within the group dynamics. The end result will be a strong caregiver group with sound goals, objectives, direction and a clear and sustainable mandate.[16]

## MATERIALS AND METHODS

### Research design

The study used qualitative paradigm; was explorative and descriptive in nature and qualitative in design; and used convenience or judgemental sampling methodologies. According to Merriam,[17] qualitative research is inductive in that the researcher builds abstractions, concepts, hypothesis, and theories from the details obtained from the field; researchers are interested in the meaning that the respondents make; that is how they make sense of their lives and lived experiences.

### Research instruments

The researcher used two almost similar interview guides as research instruments, one to collect data from the caregivers and the other to collect data from the nurses. Then two sets of data served comparative and contrast purposes. The instrument had open-ended questions to steer and guide the discussions. The results of a pilot study involving only five caregivers and one nurse had served to cleanse the instrument of its ambiguities. Nurses formed the caregivers’ supervision team.

### Sample selection criteria and procedure

All the 140 registered primary caregivers as they appeared in the community home-based care (CHBC) register and all the four CHBC nurses and their coordinator were eligible for research inclusion. The respondents’ selection criteria disregarded factors such as age and gender. While all the 5 nurses availed themselves for the interviews, 82 (59%) registered primary caregivers turned up for 10 focus group discussions. Caregivers were collected from the clinics of Kgwatlheng, Kanye Main, Mafhikana, Dada, and Mmamokhasi clinics, and from Dilolwe and Sebogo health posts that were serving them and transported to the Miracle Family Centre Church (MFCC) that formed the FGD venue. The size of the FGD groups ranged from 6 to 12. The researcher as the principal investigator was assisted by two research assistants. The research team chose to have only one session in a day in order to prepare members for the following day session.

### Ethical and legal requirements

To ensure that the study followed all the legal and ethical protocols, the researcher through the clinic heads had met the respondents well in advance, and discussed the study goals and objectives and the research process and its importance with them, their region, and the country at large. The researcher had then issued consent forms that were signed by those who were in agreement with the research process. In the forms, the researcher had promised and committed to treat the respondents with all the dignity they deserved and to maintain confidentiality and anonymity. Respondents were informed of their right and freedom to withdraw voluntarily if they wished to do so, or if they felt uncomfortable with research proceedings.[17]

The researcher was issued with a licence by the Human Research and Development Committee (HRDC) Board of the Ministry of Health upon meeting all the application conditions. The researcher had then to write a letter to the Southern District Council asking for authority to enter into the field to collect data.

### Data analysis, interpretation, and bias reduction

According to Tesch,[18] the process of data analysis is eclectic, meaning that there is no “right way” of doing it. The data analysis process started with putting together stacks and stacks of crude data that had been audio taped and transcribed. The data were sorted by the use of codes. This made the data to be arranged easily in groupings that gave rise to themes and subthemes. This formed the basis of analysis. Quotes, words, analogies, proverbs, and jotted notes were used to inform data collection, while tables and graphs were used to present the data and therefore the findings.

### Data trustworthiness and credibility

The results analyzed from the pilot study involving five caregivers and one nurse, which preceded the study, served to remove ambiguities in then instruments and had helped to focus the study more clearly. This contributed to the reduction of the study bias and improvement of data reliability and validity. The use of double translation of the instruments, that is translation from English to Setswana and then from Setswana to English by two independent translators, the two parties coming together to settle on the difference, also served to reduce data bias, resulting in increased validity as well as reliability. The fact that the two interview guides or the instruments used only differed slightly, and the two sets of responses confirmed and cross-checked each other served to strengthen data validity and reliability.

### Research domain

The data for this article were obtained from empirical research done in December 2005 and January 2006 at the Kanye village. The village, the capital town of the Bangwaketse tribe and one of the oldest in the country, had a population close to 50,000,[19] and is well endowed with five clinics and two health posts and a bigger Seventh Day Adventist (SDA) referral hospital. Though Kanye CHBC was one of those that were doing well by government standards, it was still experiencing a high death toll among the CHBC clients.[20]

## FINDINGS

### Profile of the volunteer caregivers

#### Age, gender, and educational dimension of the caregivers

Study findings indicated that the caregivers’ ages ranged from 18 to 85 years with 46 caregivers (56% of the total caregivers) being 50 years and above, with 28 of them being 60 years and above and constituting 34% of the total caregivers. The study revealed that most caregivers being women and especially those above 60 years were of low economic status and physically not strong enough to stand the care-giving demands. A larger section of the participants indicated that social support was not there and that each was working individually:

“Each one of us works on his/her own to float or sink.”

This led to their psychological disillusionment of the care-giving role. Data indicated that 72 (88%) of the caregivers had no any income to support themselves. Their psychosocial position was demonstrated by some caregivers sobbing while others broke into tears as they explained the economic and social environments in which they performed care. Caregivers wished they had working caregivers’ support groups to facilitate sharing difficulties, pain, and experiences that care giving presented to them. This was captured by a score of participants who said that

“Probably if we had caregivers’ support groups we could have addressed most of the care-giving challenges that beset us.”

In terms of literacy, 74% of the caregivers had either never been to school or had only primary level education, with only 5% of the caregivers having tertiary education. Illiteracy was found to contribute to low care productivity and poverty. This was psychologically disenabling as most of the illiterates who were also elderly had problems of accessing education on care giving, following the medical and hygiene protocol, and following disease progression of their clients. A few caregivers indicated that a caregivers’ support group, if in place, could serve as a platform from which caregivers could educate one another:

“A caregivers’ support group could open to us forums of education that will help us surmount most of the care-giving challenges such as on disease dynamism of the clients.”

Gender-based findings indicated that the Kanye program faced a serious gender-skewed dimension with 80 (98%) being women and 2 (2%) men. Caregivers complained that the process left them overwhelmed as they still had other domestic chores to attend to at home. Some made the following comments:

“It’s unfair to have only us (women) toil with care giving without the support of men. We have other important household chores to manage.”

### Caregivers’ support group as a forum for solidarity

All the Kanye study respondents confirmed that there were no caregiver support groups to encourage one another and that one was working on his/her own, either to float or sink. However, some caregivers reported that five caregivers near the Dilolwe ward were having a close working relationship with one another, sharing experiences and difficulties and encouraging one another. The relationship was, however, informal and based on good neighborliness. Caregivers pinned lack of support groups as being behind weak coordination and administration by the CHBC authorities. The below-mentioned quotes ran across the FGD room:

“One works alone in his/her small world to sink or float. There is no solidarity or good interaction.”

“Lack of caregivers’ support groups has weakened coordination and administration of the Kanye CHBC program.”

### Caregivers’ support group a forum for debriefings, learning, and experience exchange

Majority of the caregivers confirmed not being trained or given any adequate direction or supervision on care giving. All concurred that the program did not have any caregiver training schedule or program. They indicated that they were doing care giving using their God’s intuitive wisdom. They then wished that the program had support groups for the caregivers to arrange for debriefings that can result into skill development and exchange and sharing of information on care giving, and therefore empowerment of one another. A few caregivers implored that a support group has the impact of pooling the skills together, each being able to help his/her colleague depending on the level of skills and one’s comparative advantage over the other. The following words were heard from many of the respondents:

“Support groups could facilitate debriefings, and exchange and sharing of information and experiences, educating one another.”

“Support groups could be a source of empowerment of one another.”

### Caregivers’ support group as a forum for funding and mobilization of resources

About three-quarters of the caregivers confirmed that besides inadequate assistance from families, relatives, and communities in general, they did not know any adequate help from NGOs and other helping bodies. Caregivers hoped that caregivers’ support groups could act as good vehicle for fund mobilization, and a marketing platform. They said that if the caregivers’ support groups were in place, they would be able to call their political leaders and helping organizations through the forum. Some suggestions that were supported by the majority respondents were captured through the following quotes:

“We could be using the forum to mobilize resources especially from the coming businesses in Kanye and other areas.”

“Support groups could be forums that the local politicians would like to use for their political mobilizations, and in the process, we could have them mobilize resources for us.”

However, the CHBC coordinator confirming the lack of a support group as a big drawback in the Kanye programme expressed hope that the program through the financial assistance of the Global Fund Initiative would hopefully start a day care center in the Kanye program where the caregivers would be coming for information sharing and getting good and free meals. This would serve to ease their psychological burden and stress, possible depression and burn-out, and offer a room to share one’s burden with others. She suggested the need to have caregiver support groups urgently in order for caregivers to position themselves for any external assistance.

“It is urgent and critical that a support group is formed in readiness for funding from the Global Fund Initiative.”

### Caregivers’ support groups as forums for the easier administration of the program

About half of the caregivers indicated having little confidence with the way the care program was coordinated, especially the provision of the care package and the food basket. They indicated having little regard for the social workers whom they accused of taking too long to do their economic and social assessments of the clients as a leeway to the food basket. They also said that the coordinators were only in the offices and not coming to the field to know the problems the program was facing. They indicated that through a caregivers’ support group, they would be pooling their views, suggestions, and concerns that can better improve the administration and the coordination of the program. They could also be boldly facing the administration to address the innumerable challenges the program was facing. Some caregivers suggested that

“Our views and concerns could be impacting the management if we have a caregivers’ forum through a support group.”

“We could be facing the administrators and other public officers who do not work satisfactorily through a support group forum.”

### Support groups as an advocacy forum for caregivers’ rights

Kanye caregivers unanimously confirmed being excluded from any decision making involving the running of the program, with decisions taken at the management level without consulting them and yet they were the “backbone” of the program. This they considered as lack of recognition for their efforts and contribution. Caregivers were also concerned that they were not consulted or involved in making the care plan and care package to run the program. They hoped that some of these gaps could be articulated if there was a support group empowering the caregivers to articulate their feelings and concerns. Majority had the following sentiment that came out very strongly:

“We need a support group to advocate for our rights. We have been assumed for far too long by the care management.”

“It is important that we are involved in drawing a care plan as well as a care package. A support group forum could possibly facilitate this.”

### Support groups as forums of counselling

Close to three-quarters of the caregivers confirmed that they were not getting counselling to assist them deal with the dilemmas associated with care giving. This was because of poor organization and generally inadequate staffing in the care program. Caregivers suggested that a support group could easily prompt and facilitate a counselling process, especially group counselling with ease. This is because one counsellor would be able to handle a bigger number of caregivers as one entity. The following sentiment was unanimously released:

“The counselling that most of us need could be facilitated through a support group with ease.”

## DISCUSSIONS ON THE FINDINGS

### Demographics

On gender predominance of the care programs in Botswana, studies by Munodawafa,[21] Jacques and Stegling,[22] and Kang’ethe[1] found that care giving in Botswana is predominantly a female domain. Atta and Fidzani[23] indicate that over 50% of caregivers in most of the Botswana CHBC programs are women who are usually financially challenged and seriously influenced by aging and unable to follow the demands and dynamism of the care process. Since women have other domestic chores, usually to take care of families, care giving presents an extra burden. In most parts of the developing world, this has immensely contributed to feminization of poverty.[24] Because of these care-giving constraints, caregiver support groups, where caregivers will discuss care-giving concerns, challenges, and be able to encourage one another, would be critical and pivotal.

On aging, Jacques and Stegling[22] in their study in Kweneng found three caregivers who were senile and not able to discharge care-giving tasks. McDonnel *et al*.[25] warn against leaving care giving in the hands of the elderly who are vulnerable. They advise that failure to have them helped might lead to collapse of the informal caring systems that continue to be relied upon especially in the developing world. However, the decline in the caring strength can be explained by the second law of thermodynamics that indicates constant loss of energy in the body as aging takes toll.[26]

### Recommendations

Education and advocacy targeting to achieve sharing of roles by both gender in care giving need to encouraged by Government, civil society bodies and communities generally.

A support group would be very important to be used as a platform of education and advocacy to win men and the young into care giving duties.

On the low level of education among the caregivers, other studies in Maun by Phorano *et al*.[27] complemented by those of Kang’ethe[1] in Kanye found that most caregivers were of low educational level. Low education has a bearing toward the quality of care giving as HIV/AIDS and care giving continue to be complex and dynamic calling for more understanding. Issues of drug administration, hygiene, and palliative care become friendly to those whose educational capacity is adequate. If the study area had caregiver support groups, arranging with care authorities and possibly government for continuous and on the job training could possibly be achieved.[28]

The government and care authorities should institute a continuous and on the job training for the caregivers.

Kanye caregivers wished for a caregivers’ support group forum to achieve solidarity and help one another. The need and importance of the caregivers helping one another fulfills the call embedded in the 1995 World Health Organization’s AIDS Day theme of *shared rights and shared responsibilities*,[29] whereby all involved in the care process need to feel duty bound to assist one another for the care process to realize huge dividends. This solidarity is likely to strengthen the help system and make the program achieve its goal of being a safety valve to the congested health facilities.[30]

According to NAB,[5] a caregiver support group is likely to help members in the following ways: installation of hope, making what seems unique, similar, or identical experience of another group member; forum for information exchange through group discussion; and altruism. Support groups are very crucial and relevant to caregivers as they can help to lessen the isolation of caregivers and normalize their journey of grief.[5,31]. The Zululand Hospice home-based care program’s support group in South Africa, for example, sees caregivers holding meetings at which educational talks, prayers, handwork, singing, counseling and teachings, and members’ performance assessments are done.[32]

Care managers need to mobilize the formation of caregivers’ support groups. They need to borrow ideas from other districts or other countries where such support groups are working. Caregivers are also challenged to mobilize themselves and have even informal helping systems with one another like the caregivers of the Dilolwe ward in the Kanye village.

The researcher suggests that caregivers’ support groups could be important for they could serve as a forum for debriefings. According to Uys and Cameron,[9] a debriefing is a formal meeting, done individually or in small groups usually after stressful incidents for the purpose of dealing with the emotional residuals of the event. According to Uys and Cameron,[9] NAB,[5] and websites like http:ourworld. compuserve.com/homepages/johndweaver/debrief.htm,[33] debriefing helps restore confidence and strength in a caregiver so that he/she can continue working in a stressful environment successfully. Debriefing involves sharing thoughts, feelings, emotions, experiences, pains, and memories one may be going through, the ultimate goal being to afford successful recovery; restoration of one’s emotional and psychological wellbeing; and normalizing living. Since the Kanye program has had a high death toll, there are possibilities the death toll could be brought down due to essential information and education infiltrating most of the caregivers.[34]

A caregivers’ support group could be a forum for fund raising and resource mobilization. Although the researcher knew of some bodies such as Standard Bank and Barclays Bank giving an assistance package especially to the orphans and the caregivers and their clients, it appeared that the help package was insignificant in that the caregiver research participants did not know or had not benefited from such help gesture. However, the fact that caregivers were working alone with no any organised support fast miss such help gesture or the help could be unfairly be disseminated especially if the program suffers from poor administrative and coordinative challenges. In Zimbabwe, the Chirumhanzu CHBC program has six caregivers’ support groups whose goal is to mobilize resources and increase the economic self-reliance of members by teaching them sewing (by hand and machine) and gardening skills. Some are also involved in raising chickens together.[31,35]

From the researcher’s point of view, a support group can facilitate the administrative and coordinative aspects of the CHBC program. According to Van Dyk,[36] a support group is a structure where people meet on a regular basis to talk about their difficulties or simply to relax and enjoy each other’s company. It offers an opportunity to give and receive, to share, to listen to, and to witness others’ experiences and vice versa without being judged, blamed, or isolated. It can also be a robust forum for administrative as well as coordinative purposes.

Lack of caregivers’ involvement in decision making of the program had a serious bearing toward their morale and ownership of the program. A support group could be used as a front to express caregivers’ rights, their complaints, and grievances. According to Uys and Cameron,[9] it should be the responsibility of CHBC health workers to go to the homes and help the caregivers in the presence of the client where possible, and to show or help develop the care plan and care package. Uys and Cameron[9] advise that involving the caregivers in developing and making the care plan is very important. It also heralds a strengthened care ownership. This has an advantage of making the client cooperative, and hence reduces any chances of caregiver–client conflict in the care process.

Using the support group forum, caregivers would also be in a position to advocate and demand adequate service provision of facilities such as protective clothing. They would for instance be interested to question whether in an eventuality that an injury (viral contraction) were to happen in the process of their care giving process, they would be considered for redress compensation just like their health personnel colleagues in health facilities. This is because they are doing the same job, themselves contributing immensely to the continuum of care[27,37].

However, the care-giving process has been hit by immense challenges that require advocacy. They include inadequate or irregular provision of care package facilities such as protective clothing in the health centers, amid cultural dilemmas pertaining to their use; caregivers’ ignorance and inadequate care-giving knowledge and skills among others; and many caregivers being at risk of succumbing to HIV/AIDS through a contagion.[1] However, studies done by Rampa *et al*.[28] found that due to caregivers’ high illiteracy rate and poor education in health matters, infected patients are treated at home without proper established medical care and a well-structured home-based care mechanism that embraces professional practice standards, as a result of which many family members have been infected with the virus unknowingly. The role, therefore, of support groups in forming and facilitating a strong advocacy forum to articulate caregivers’ rights is critical and significant. A caregivers’ support group is also likely to foster education of the caregivers as it is easier to offer education to an organized group.

It is recommended that the Government of Botswana and care authorities increase their commitment to and surveillance of care programs, identify the operational constraints such as lack of support groups, and work toward mobilizing their formation.

A caregiver’s support group can be a good forum for counseling. According to Nurses Association of Botswana (2004),counseling is a one-to-one (or one-to-many) relationship between a counselor (the expert) and the client/s with a problem or concern. However, the most important virtue a counselor can offer to a client is presence and concern. He/she can only facilitate the process by being there, by listening and being nonjudgemental, and by assuring the clients that they are not going crazy and that the acute pain they are experiencing will not last forever. Counseling can help caregivers emotionally cope with the dying and bereaved people and help relatives and colleagues handle other people’s emotional release.[9,5]

## CONCLUSION

The role of caregivers’ support groups in bringing the caregivers together; fostering their counseling; debriefings, education, and sharing information and experiences; sharing their pains, concerns, and frustrations cannot be overemphasized. As a group, it is easier for the caregivers to bolster one another and to come out with strategies to seek assistance from various avenues; be recognized and be respected by authorities; possibly prompt recognition of the group and its role; and possibly increase its horizon of assistance. A support group offers hope, consolation and instills confidence in members of the group. It is important that all care-giving programs in Botswana have caregivers’ support groups. They are likely to increase caregivers’ morale and productivity.
